# Protein profile of different cellular fractions from *Mycobacterium tuberculosis* strains after exposure to isoniazid

**DOI:** 10.1016/j.dib.2019.103953

**Published:** 2019-04-25

**Authors:** Luisa María Nieto Ramirez, Carolina Mehaffy, Karen M. Dobos

**Affiliations:** a Department of Microbiology, Immunology and Pathology, Colorado State University, Fort Collins, CO, USA; b Universidad Santiago de Cali, Cali, Colombia

## Abstract

Different biochemical studies looking for the effect of INH on the physiology of *Mycobacterium tuberculosis (Mtb)* have been conducted. Here, we present a detailed analysis, looking at the protein variation in the *Mtb* cell due to exposure of sub-inhibitory concentrations of INH, evaluating three different variables: cellular fraction, genetic lineage, and INH phenotypic profile. Mass spectrometry analysis demonstrated that the most significantly affected cellular fraction was the membrane and the INH resistant strains showed the highest number of proteins altered when they were exposed to INH. Raw data are available via ProteomeXchange with identifier PXD007588.

**Table:** Specifications table [*Please fill in right-hand column of the table below*.]

Subject area	*Microbiology*
More specific subject area	*Mycobacteriology and proteomics.*
Type of data	*Tables, figures, text file, mass spectrometry data.*
How data was acquired	*Mass spectrometry and Western blot*
Data format	*Raw and analyzed.*
Experimental factors	*Mtb strains were cultured with and without different concentrations of isoniazid depending on the susceptibility to the drug.*
Experimental features	*We cultured different* Mtb *strains in liquid media to obtain cellular biomass that after physical and chemical methods was fractionated into cytosol, membrane, cell wall, and secreted proteins. The resulting samples were standardized to the same amount of total purified protein for digestion with trypsin followed by nanoLC-MS/MS analysis as previously described*[1]. *Using the platforms* Sorcerer2™ and SEQUEST, *the resulting spectra were analyzed using the identification criteria previously defined by Nieto* et al. *and the* Mtb *strain H37Rv Tuberculist database*[1]. *Finally, western blot analysis was conducted for five* Mtb *proteins to confirm mass spectrometry findings.*
Data source location	*Fort Collins, Colorado.*
Data accessibility	*Raw data of this article are available* via *ProteomeXchange with identifier PXD007588*
Related research article	*Nieto R LM, Mehaffy C, Islam MN, Fitzgerald B, Belisle J, Prenni J* et al. *Biochemical characterization of isoniazid resistant. Mol Cell Proteomics.*2018*. Epub 2018/05/29.*https://doi.org/10.1074/mcp.RA118.000821*. PubMed PMID: 29,844,232*[1]*.*

**Table:** 

**Value of the data** • *This data shows the first characterization of changes at the proteome level after the exposure of Mycobacterium tuberculosis (Mtb) to isoniazid (INH), looking at different subcellular levels (cytosol, cell wall, membrane and secreted proteins) and using a very sensitive methodology: label-free quantitative proteomics (by LC-MS/MS), complemented* [1] *with western blot (WB).* • *This data brings upfront the importance of studying the bacterial proteome of laboratory and clinically relevant genotypes and phenotypes (drug-susceptible and drug-resistant to INH), when* Mtb *is in contact with one of its more potent drugs.* • *Common protein variations commonly found in both INH-related phenotypes of Mtb (INH susceptible and resistant), were associated with lipid biosynthesis, central carbon metabolism, lipoproteins, and the INH activator (KatG).* • *Reduced levels of some proteins presented here were also revealed in our previous study that compared clonal strains of Mtb after acquisition of INH resistance* [1], *which could be reflecting the steps towards bacterial adaptation to tolerate INH.* • *The metabolic routes and the specifically identified proteins could serve as potentially complementary drug targets, in the design of more rationalized anti-TB therapy using INH, which is still one of the best treatment options for patients suffering from TB.*

## 1 Data

**Fig. 1:**
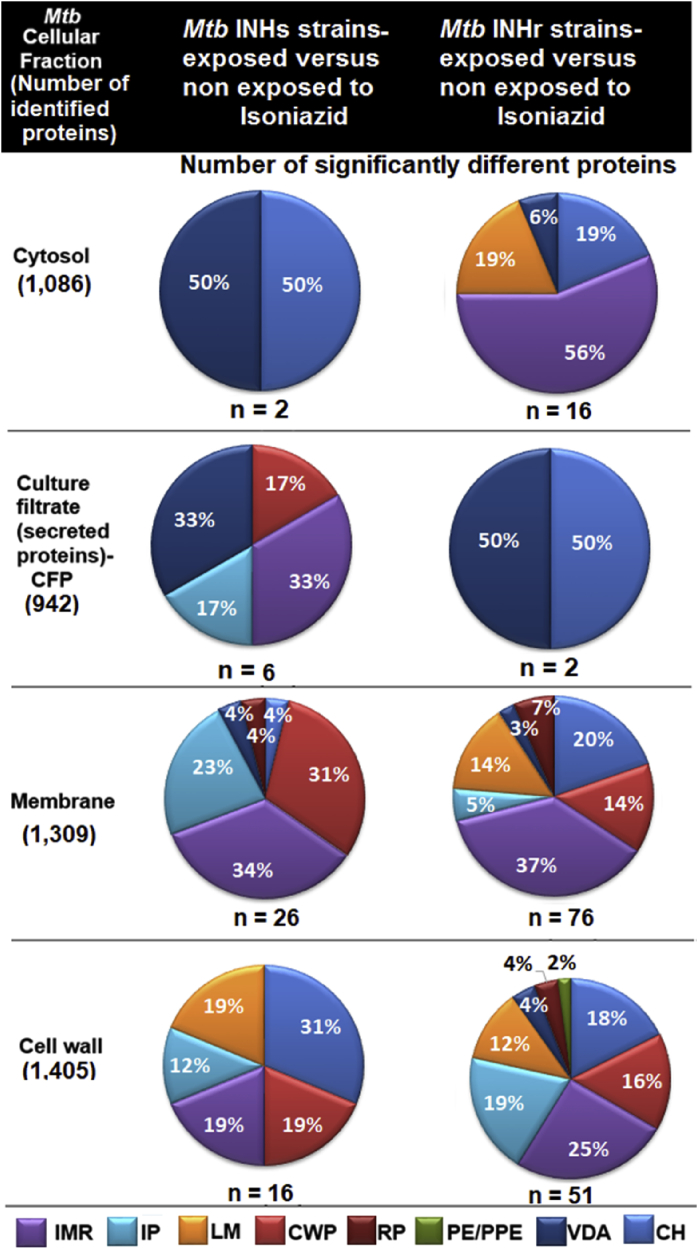
Distribution of the proteins with significantly different levels (*t*-test, corrected with Benjamini-Hochberg, with Q = 10%) for each comparison group (selecting commonly altered proteins in the INHs and INHr strains separately). Proteins are grouped according to their functional category: IMR: Intermediary metabolism and respiration, IP: Information pathways, LM: Lipid metabolism, CWP: Cell wall and cell wall processes, RP: Regulatory proteins, VDA: Virulence, detoxification and adaptation, CH: Conserved hypothetical.

Data from each of the four protein fractions (cytosol, membrane, cell wall, and secreted proteins) collected for the three biological replicates of the four strains (clonal pairs: T genotype INHs and INHr, as well as H37Rv and its M1A mutant) were analyzed. The total number of proteins identified at each cellular compartment as well as the number of significantly different proteins that were altered in both INHs and INHr strains exposed to INH, after Benjamini-Hochberg (BH) correction (with false discovery rate-Q of 10%)) is presented in Fig. 1. Overall, the most significantly altered cellular fraction and functional category were the membrane and intermediary metabolism and respiration respectively (Fig. 1). There were 10 significantly different proteins demonstrating levels that changed similarly across the different fractions evaluated; none of the soluble fractions (cytosol or secreted fractions) exhibited significantly different proteins after this BH correction (Table 1, Fig. 1). Using a less conservative Q value of 20% in the BH correction, the number of significantly different proteins increased to 42. These changes were observed regardless of the genetic background of the bacteria or the organism's susceptibility to INH. The proteins presented in these comparisons were at least found in two different cellular fractions with the same trend. Although we could observe significantly different proteins in each pair comparison individually (data not shown) using a Q value of 5%, none of the proteins resulted in significantly different abundances across all the pair wise comparisons tested at each fraction (Table 1).

**Table 1:**
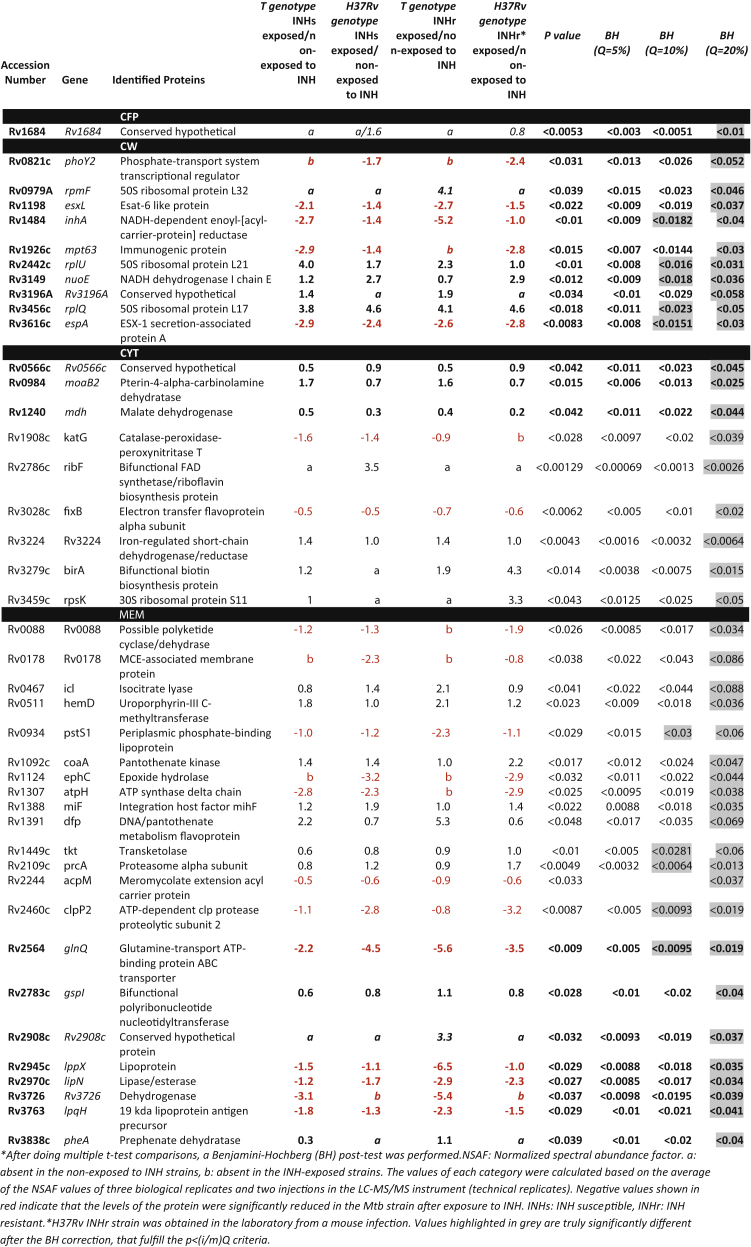
Significantly altered proteins in *Mtb* strains after exposure to INH classified according their susceptibility profile to INH and genetic background pair comparisons tested with *t*-test, corrected with Benjamini-Hochberg (BH)^a^ with different false discovery rates (Q) values. $\text{Log}2\;\text{f}\;\text{old}\;\text{change}\;\frac{NSAF\;\text{exp}osed\;to\;INH}{NSAF\;non-\text{exp}osed\;to\;INH}$.

**Fig. 2:**
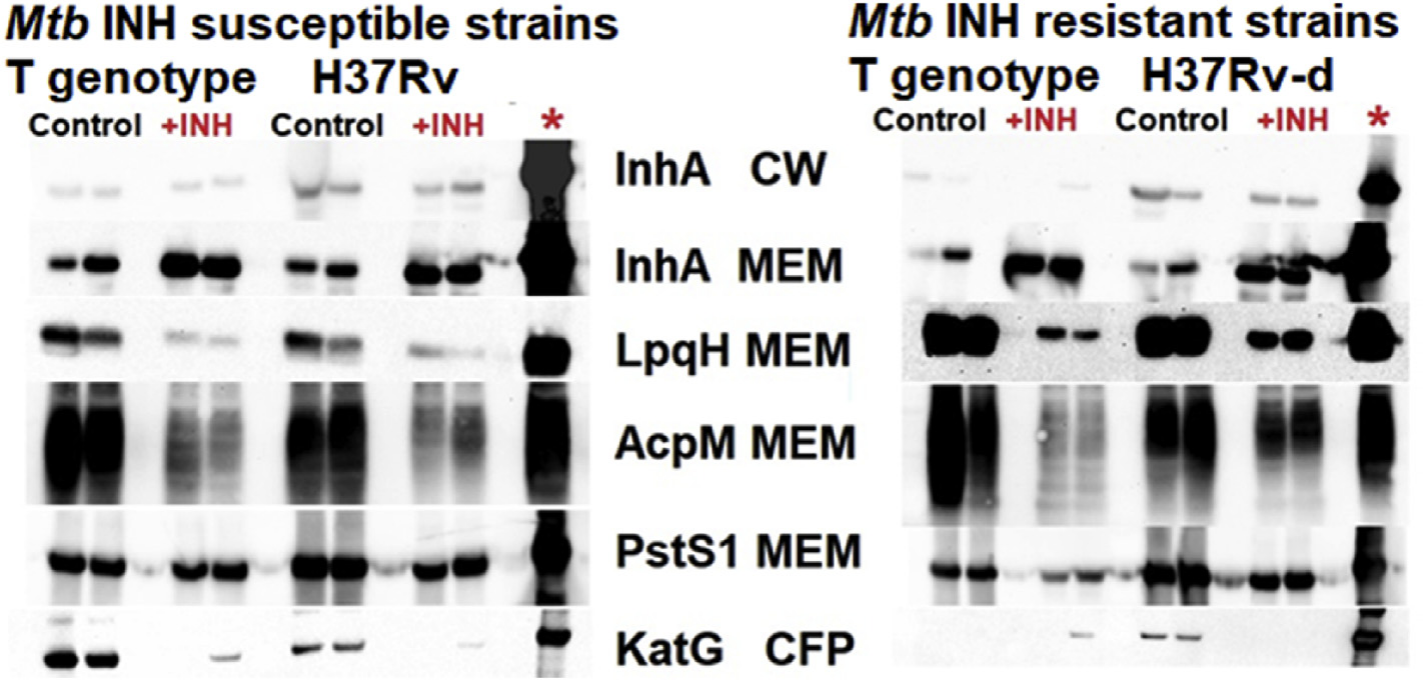
Western blot confirmation of some proteomic results. Two biological replicates of Mtb strains were analyzed in each group compared. Each pair of biological replicates of each condition (control and exposed to INH (+INH)) were separated by an empty well. INHs strains were exposed to 0.05 μg/mL and INHr strains were exposed to 0.2 μg/mL of INH. H37Rv-d indicates an INH resistant strain obtained from the reference strain H37Rv in the laboratory, after exposing a Mtb-infected mouse to INH. The last well in each gel (*) corresponds to the positive control, 0.5 μg recombinant InhA, and 5 μg MEM obtained from H37Rv reference strain for the other proteins.

InhA and PstS1 were commonly found in the analysis of all pair wise comparisons using a Q of 10% in BH correction (Table 1); Western blot (WB) analysis was used to confirm this finding, since antibodies were available for confirmation (Fig. 2). In addition, there were three additional proteins: LpqH, AcpM and KatG that exhibited significant differences, albeit only using a Q of 20% in BH correction (Table 1) for which antibodies were also available and thus used to confirm this finding (Fig. 2). InhA levels were significantly reduced in the cell wall when the *Mtb* strains were exposed to INH according to the LC-MS/MS results (Table 1); this was confirmed through WB analysis (Fig. 2). Interestingly, the levels of this protein were strongly increased in the membrane fraction of all the strains evaluated as demonstrated through WB (Fig. 2). LC-MS/MS values also showed increased InhA levels in the membrane fraction in all the comparisons, however this difference was only significant when H37Rv was exposed to INH (p = 0.00064, fold change = 3.8). Additionally, WB analysis of LpqH and AcpM confirmed the LC-MS findings demonstrating that their levels decreased when the strains were exposed to INH (Fig. 2, Table 1). Finally, among the soluble proteins, KatG was significantly reduced in all strains when they were exposed to INH (Table 1). This could be corroborated through WB analysis of the soluble, secreted fraction (Fig. 2).

## 2 Experimental design, materials, and methods

***Sample preparation.*** Two group of *Mtb* strains were used in this study, one pair, belonging to the T genotype, was clinically-isolated while the other pair corresponded to the reference strain H37Rv and its isogenic INH resistant counterpart [1]. H37Rv belongs to the Euro-American lineage. In each group, there was one INH susceptible (INHs) and one INH resistant (INHr) strain obtained in the clinical or the laboratory setting. In both cases, the INHr strain was isolated after the parental *Mtb* strain was exposed to the drug. All INHs and INHr strains were cultured in 100 mL of Glycerol Alanine Salts (GAS) media and corresponded to the control group.

For the experimental and control condition (with and without INH respectively), the bacterial cultures were incubated at 37 °C in constant agitation for three weeks. The concentration of INH used for the experimental condition (exposed to the drug) was previously determined *in vitro,* evaluating the growth on 7H11 media at different INH concentrations in both INHs and INHr *Mtb* strains. The test was performed following the proportion method in agar [2], testing concentrations of INH ranging from 0.025 μg/mL to 1 μg/mL. All the bacterial cultures in the experimental condition were in contact with INH from the first culture (frozen stock to 7H11 plates) up to culture in the liquid GAS media, using a concentration of INH of 0.05 μg/mL for the INHs strains and 0.2 μg/mL for the INHr strains.

After the incubation period, *Mtb* cells were harvested by centrifugation at 3000×*g* for 20 minutes and the culture supernatants were sterilized using a 0.2 μm filter. Prior to bacterial lysis and cellular fractions preparation, cells were inactivated by gamma irradiation and inactivation confirmed by the Alamar Blue Assay following the manufacturers’ protocol. In order to maintain the consistency in the analytical conditions, steps from protein purification, digestion, clean up, LC-MS/MS analysis and data base searching was performed as was described in our previous work [1]. Briefly, the CFPs were concentrated from 100 mL to approximately 2 mL using a Millipore^TM^ Amicon^TM^ Bioseparation Stirred Cell with a 3-KDa mass cutoff membrane (Millipore). Further buffer exchange with 10 mM Ammonium bicarbonate was performed using Amicon Ultra-15 centrifugal filter units with a 3-kDa molecular mass cutoff.

The cell pellet of each biological replicate sample was suspended in breaking buffer (1 mM EDTA-PBS supplemented with 60 μg of DNase and 60 μg of RNase and one tablet of cOmplete™ Protease Inhibitor Cocktail (sigma-aldrich) per 50 mL of buffer). Cells were subjected to lysis, using 10 cycles of probe-sonication (90 seconds on and 30 seconds off) maintaining cells in ice, as previously described [3], [4]. All the residual intact cells and other cell debris were removed by centrifugation at 3000×*g* for 15 min at 4 °C. After this, *Mtb* cellular fractions including membrane (MEM), cytosol (CYT) and cell wall (CW) were obtained by continuous cycles of ultracentrifugation, as described by Lucas et al. [5]. Briefly, CW fraction was separated from MEM and CYT by centrifugation at 27,000×*g* at 4 °C for 1 hour. The resulting supernatant was subjected to two consecutive ultracentrifugation cycles at 100,000×*g* during 4 hours at 4 °C, obtaining the MEM in the pellet and CYT in the supernatant after each cycle. All the resulting proteins were resuspended in 10 mM ammonium bicarbonate. Before the protein digestion, the CW fraction was delipidated as described elsewhere [4]. Each fraction was qualified for total protein content and marker proteins (where applicable), per standard qualification criteria used to qualify and provide similar biological reagents to the mycobacteria research community through BEI resources (https://www.beiresources.org/About/QualityControl.aspx).

Total protein concentration of all subcellular fractions and CFPs was measured using the bicinchoninic acid method (BCA) (Thermo Scientific™Pierce™BCA Protein Assay). In-solution trypsin digestion of 30 μg of each protein sample using ProteaseMax surfactant was performed as described previously [4], followed by a final desalting step using Pierce^®^ C-18 spin columns (Thermo Scientific) before LC-MS/MS analysis was performed as previously described [1]. The resulting peptides were diluted in solvent A (0.1% formic acid, 3% ACN in HPLC water) for the LC-MS/MS analysis.

***LC-MS/MS***. One microliter (0.5 μg) of digested peptides from subcellular fractions and CFPs were randomly injected in duplicate using the Orbitrap Velos MS coupled with nano-HPLC instrument (Thermo Scientific). Each sample was injected using an EASY nanoLC-II system (Thermo Scientific, San Jose, CA). Peptides were purified and concentrated using an on-line enrichment column (EASY-Column, 100 μm ID × 2 cm ReproSil-Pur C18). Subsequent chromatographic separation was performed on a reverse phase nanospray column (EASY-Column, 3 μm, 75 μm ID × 100mm ReproSil-Pur C18) using a 90-min linear gradient from 5% to 45% solvent B (100% Acetonitrile, 0.1% formic acid) at a flow rate of 400 nL/min. Peptides were eluted directly into the mass spectrometer (Thermo Scientific Orbitrap Velos). The instrument was operated in Orbitrap-LTQ mode where precursor measurements were acquired in the Orbitrap (60,000 resolution) and the tandem MS/MS spectra (top 20) were acquired in the LTQ ion trap with a normalized collision energy of 35%.

***Database searching***. Tandem mass spectra raw data were converted to mzXML files using ProteoWizard (MSConvert version 3.0) [6]. All MS/MS samples were analyzed using Sorcerer2™ integrated data analysis platform (Sage-N Research, Milpitas, CA, version 5.0.1) and SEQUEST (Thermo Fisher Scientific, San Jose, CA, USA; version v. 3.5). SEQUEST was set up to search the *Mtb* strain H37Rv Tuberculist database [7] including all reverse entries as decoys (7992 entries) with trypsin as the digestion enzyme and up to two missed cleavage sites. SEQUEST was searched with a fragment ion mass tolerance of 1.00 Da and a parent ion tolerance of 20 ppm. Oxidation of methionine (15.99 amu) and carbamidomethylation of cysteine (57.02 amu) were specified in SEQUEST as variable modifications.

***Criteria for protein identification.*** Scaffold (version Scaffold_4.5.3, Proteome Software Inc., Portland, OR) was used to validate MS/MS based peptide and protein identifications. Peptide identifications were accepted if they could be established at greater than 95.0% probability by the Scaffold Local FDR algorithm. Protein identifications were accepted if they could be established at greater than 99.0% probability and contained at least 2 identified peptides. Protein probabilities were assigned by the Protein Prophet algorithm [8]. Proteins that contained similar peptides and could not be differentiated based on MS/MS analysis alone were grouped to satisfy the principles of parsimony. Differences between protein abundances, expressed as normalized spectra abundance factors (NSAF values) among the two different conditions (exposed versus non-exposed to INH) in each pair were tested by two tailed Student's *t*-test, resulting in four pair comparisons. The subsequent Benjamini-Hochberg post-test correction was applied. All p values less than (i/m)Q were considered significant, where *i* is the rank, *m* is the total number of tests for each cellular fraction analyzed (the total number of proteins identified at each fraction), and Q is the false discovery rate that was set to 5% and 20%, as recommended by Diz et al.,. [9], including an intermediate value of 10%. The mass spectrometry proteomics data have been deposited to the ProteomeXchange Consortium via the PRIDE partner repository [10] with the dataset identifier PXD007588 and 10.6019/PXD007588.

***Western blot (WB) assays***. Validation of the InhA, LpqH, AcpM, PstS1 and KatG abundances among the different strains and experimental conditions were confirmed by WB. These proteins were selected depending on the antibody availability in the laboratory. Primary antibodies were obtained from different sources that included: anti-LpqH, -PstS1 and -KatG from BEI (https://www.beiresources.org/). Anti-InhA was provided by Dr. John Spencer from Colorado State University. Rabbit polyclonal anti-AcpM was produced in a previous study described by Nieto et al.*,.* [1].
